# Heart Failure with Mildly Reduced Ejection Fraction—A Phenotype Waiting to Be Explored

**DOI:** 10.3390/jcdd11050148

**Published:** 2024-05-09

**Authors:** Anugrah Nair, Lukah Q. Tuan, Natasha Jones-Lewis, Deep Chandh Raja, Jenish Shroff, Rajeev Kumar Pathak

**Affiliations:** 1 Department of Cardiac Electrophysiology, Canberra Heart Rhythm Centre, Canberra, ACT 2605, Australia; anugrah@chrc.net.au (A.N.); lukah.tuan@canberraheartrhythm.com.au (L.Q.T.); natasha.jones-lewis@canberraheartrhythm.com.au (N.J.-L.); jenish.shroff@canberraheartrhythm.com.au (J.S.); 2 ANU College of Health and Medicine, Australian National University, Acton Campus, Canberra, ACT 0200, Australia; deepchandh@gmail.com

**Keywords:** heart failure with mildly reduced ejection fraction, cardiac imaging, myocardial scar, catheter ablation, intra-cardiac defibrillator, physiological pacing

## Abstract

Heart failure (HF) presents a significant global health challenge recognised by frequent hospitalisation and high mortality rates. The assessment of left ventricular (LV) ejection fraction (EF) plays a crucial role in diagnosing and predicting outcomes in HF, leading to its classification into preserved (HFpEF), reduced (HFrEF), and mildly reduced (HFmrEF) EF. HFmrEF shares features of both HFrEF and HFpEF but also exhibits distinct characteristics. Despite advancements, managing HFmrEF remains challenging due to its diverse presentation. Large-scale studies are needed to identify the predictors of clinical outcomes and treatment responses. Utilising biomarkers for phenotyping holds the potential for discovering new treatment targets. Given the uncertainty surrounding optimal management, individualised approaches are imperative for HFmrEF patients. This chapter examines HFmrEF, discusses the rationale for its re-classification, and elucidates HFmrEF’s key attributes. Furthermore, it provides a comprehensive review of current treatment strategies for HFmrEF patients.

## 1. Introduction

**Figure 1:**
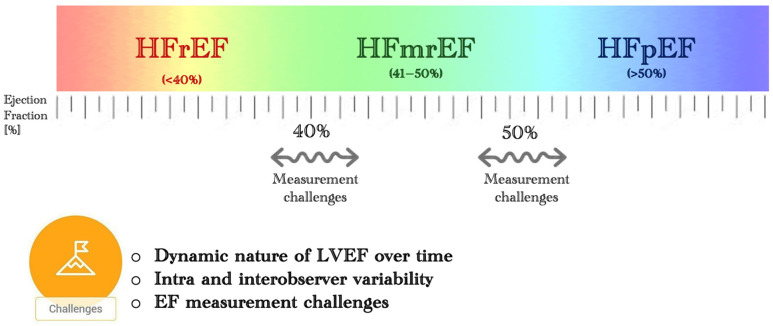
Demonstration of the spectrum of heart failure and the challenges when assessing LVEF. This spectrum includes HFmrEF (heart failure with mildly reduced ejection fraction), HFpEF (heart failure with preserved ejection fraction), and HFrEF (heart failure with reduced ejection fraction).

Heart failure (HF) is growing in prevalence and represents a significant global health challenge. Despite the availability of effective treatments, HF patients face poor prognoses, with high rates of hospitalisation and annual mortality ranging from 10 to 35% across various population-wide registries [1,2]. Advanced HF carries even higher mortality rates. The left ventricular (LV) ejection fraction (EF), assessed typically through echocardiography, is pivotal for HF diagnosis (Figure 1), prognosis, patient classification, and treatment decisions. The 2021 European Society of Cardiology (ESC) guidelines categorise HF into three types, based on LVEF: preserved (HFpEF; EF > 50%), reduced (HFrEF; EF < 40%), and mildly reduced (HFmrEF; EF 41–49%) [3]. Research indicates that HFmrEF not only shares characteristics of both HFrEF and HFpEF but also exhibits distinct features [4,5].

### The Emergence of Heart Failure with Mildly Reduced Ejection Fraction

Since the 1980s, EF has served as the primary diagnostic, classificatory, and risk-stratifying tool for HF. Historically, HF classification based on EF has delineated two distinct groups: HFrEF and HFpEF. The 2013 American College of Cardiology/American Heart Association (ACC/AHA) guidelines categorised patients with an EF between 41 and 49% as potentially bordering HFpEF, distinguishing them from those with HFrEF [6,7,8,9,10]. Subsequently, in 2016, the ESC introduced a category between the two traditional entities, termed “HF mid-range EF”; the more contemporary ESC guidelines in 2021 define this as being the same as HF with mildly reduced ejection fraction (HFmrEF) [11,12].

The intention of the guidelines committee was not to abruptly introduce new clinical entities with specific therapeutic and morphological backgrounds, but rather to encourage dedicated research into the “underlying characteristics, pathological aspects, and therapeutic features of the patient population”. According to the 2021 ESC guidelines [3], the diagnosis of HFmrEF is based on the presence of clinical symptoms and signs [13,14], alongside an LVEF ranging from 41 to 49%. Additional criteria such as structural and functional cardiac abnormalities (LV hypertrophy and/or enlarged LV and/or diastolic dysfunction) and elevated natriuretic peptides further support the diagnosis of HFmrEF. 

## 2. Epidemiology

In Western nations, the annual incidence of HF varies from 1 to 9 cases per 1000 individuals, constituting approximately 2% of the population. The prevalence of HFmrEF among all HF patients is estimated to range from 10 to 25%. In extensive longitudinal studies conducted within community-based cohorts, the incidence of HFmrEF was found to be only 6.7 per 10,000 population per year. In comparison, the incidence of HFpEF and HFrEF were 26.9 and 34.9 per 10,000 population per year, respectively, with predictors of HF events remaining consistent across the EF spectrum [15]. In the ESC-HF-LT registry, 24% of patients presented with HFmrEF, while in the Swedish HF registry, 21% of patients presented with HFmrEF. Reports from Asian, New Zealand and North American regions indicate a slightly lower prevalence of HFmrEF [16]. 

## 3. Pathophysiology

**Figure 2:**
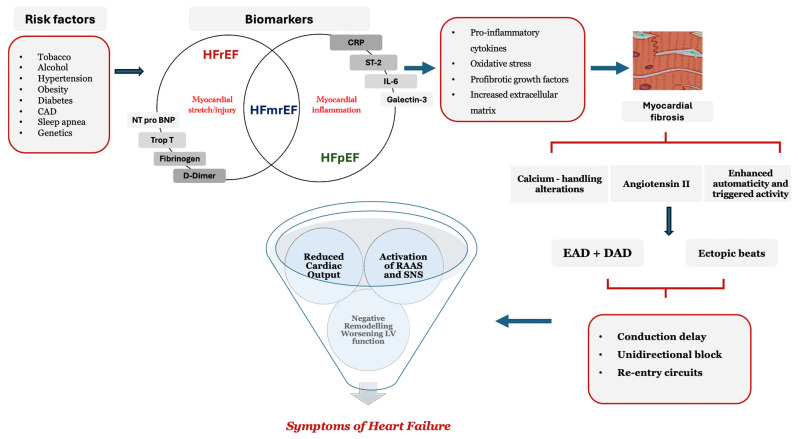
Flowchart demonstrating the pathophysiology seen in heart failure with mildly reduced ejection fraction. There exists an overlap between risk factors, myocardial stretch/injury, and myocyte inflammation in terms of the pathogenesis in HFmrEF. The pathophysiological mechanisms lead to alterations in calcium handling, with neurohormonal activation leading to LV negative remodelling and HF. Abbreviations: CAD, coronary artery disease; NT-pro BNP, N-terminal pro-B-type natriuretic peptide; Trop, troponin; ST-2, suppression of tumorogenicity-2; IL-6, interleukin-6; CRP, C-reactive protein; EAD, early after depolarisations; DAD, delayed after depolarisations; RAAS, renin angiotensin-aldosterone system; SNS, sympathetic nervous system.

Common fundamental pathophysiological processes (Figure 2), such as endothelial dysfunction, cardiac injury, myocardial dysfunction, and the activation of neurohumoral pathways, contribute to the pathogenesis of all HF subgroups [17,18,19]. The BIOSTAT-CHF study revealed that HFmrEF patients display biomarker profiles suggestive of cell proliferation and metabolism, resembling those observed in HFrEF (e.g., growth differentiation factor 15, N-terminal pro-B type natriuretic peptide, interleukin 1 receptor). Additionally, they resemble profiles indicating inflammation and extracellular matrix reorganisation, like those seen in HFpEF (e.g., beta2-ethyl-2 catenin) [20,21,22,23].

## 4. Evaluation

### 4.1. Cardiac Imaging 

#### 4.1.1. Echocardiography

LVEF is the cornerstone for HF classification. Previous studies have shown that a 10% reduction in EF escalates the risk of mortality by 39% [24]. However, once the LVEF exceeds 45%, the risk of final endpoint events stabilises, underscoring the limitations of LVEF in predicting future occurrences. Global longitudinal strain (GLS) emerges as a more reliable parameter. 

Myocardial strain reflects alternations in tissue deformation during each cardiac cycle and proves valuable for the early detection of LV dysfunction. Furthermore, GLS outperforms LVEF as a predictive indicator of HF readmissions and malignant arrhythmias [25,26]. Staton et al. found that GLS remains an independent prognostic factor for all-cause mortality, even in patients with an LVEF above 35%, providing additional prognostic insights beyond those derived solely from LVEF [27].

#### 4.1.2. Cardiac MRI

Myocardial fibrosis can result from various pathological processes and is linked to unfavourable clinical outcomes. Cardiac magnetic resonance imaging (CMR) offers a non-invasive means of assessing cardiac structure, function, and tissue characterisation, including late gadolinium enhancement (LGE), which is crucial for detecting myocardial fibrosis. However, LGE analysis is primarily qualitative and semi-quantitative. The T1 mapping technique provides a novel approach for quantitatively analysing myocardial fibrosis, offering non-invasive and symmetric evaluation. Diseases causing myocyte edema can elevate native T1 values [28]. 

A newer CMR sequence known as feature tracking (FT) measures LV strain (longitudinal, circumferential, and radial) using steady-state free precession image tracking. CMR-FT is quick, semi-automatic, does not require additional scanning or sequences, and reduces the processing time. Raja DC et al. studied scar localisation in 19 patients using a CMR-FT strain and electro-anatomical scar voltage map. They concluded that the abnormal myocardial strain detected using the CMR-FT method is more closely related to electrical abnormalities than the conventional LGE detected by CMR. The localisation of low voltage zones with CMR-FT strain has better concordance than LGE. Thus, CMR-strain readings can inform the operator about specific regions of substrate abnormalities during a ventricular tachycardia (VT) ablation procedure, especially in the absence of an LGE scar [29]. 

### 4.2. Biomarkers

#### 4.2.1. N-Terminal Pro-B-Type Natriuretic Peptide

HF induces pressure overload in the cardiac chambers, leading to the increased secretion of B-type natriuretic peptide (BNP). N-terminal pro BNP serves as a diagnostic marker for patients with HFrEF and HFpEF [30]. In an observational study involving 9847 outpatients with HF [31], HFmrEF patients showed an average NT-pro BNP level of 1540 pg/mL. Patients with higher NT-pro BNP values have twice the risk of all-cause mortality and HF re-hospitalisation compared to those with lower levels (HR: 1.48; 95% CI: 1.36–1.61).

The ESC 2021 and 2022 ACC/AHA guidelines highlight the importance of BNP/NT-proBNP testing in ruling out HF in the emergency setting, as well as recommend its measurement for risk stratification and the establishment of a prognosis [31].

#### 4.2.2. Cardiac Troponin

The cardiac troponin complex (cTn) consists of 3 subunits found within the fibres of striated muscle: troponin T (TnT), troponin I (TnI), and troponin C (TnC). TnT acts as a linking protein between the troponin complex and tropomyosin, while TnI regulates the interaction between actin and myosin. Additionally, simultaneous measurements of high-sensitivity cardiac troponin T (hs-cTnT) and NT-pro BNP can improve the identification of patients at high risk. Furthermore, even in patients with normal NT-pro BNP levels, hs-cTnT remains independently associated with the occurrence of adverse events, suggesting that hs-cTnT may offer additional prognostic information [32,33]. 

#### 4.2.3. Other Biomarkers

Galectin-3 (Gal-3) is a soluble glycoprotein with the capacity to bind to galactosides. It can stimulate myofibroblast proliferation and collagen accumulation, thus playing a role in myocardial fibrosis [34].

Growth differentiation factor 15 (GDF-15), also referred to as macrophage inhibitory cytokine-1 (MIC-1), belongs to the transforming growth factor superfamily and is typically expressed in a long-term manner. Under normal physiological conditions, GDF-15 expression in human tissues, except for the placenta, is minimal. However, under pathological conditions, its expression in human tissues can be regulated by the p53 gene in response to inflammation, hypoxia, and oxidative stress. Fernandez et al.’s study compares the role of GDF-15 in HFpEF and HfmrEF patients. They found that while there are no significant differences between HFpEF and HFmrEF, GDF-15 independently predicts all-cause mortality in both groups. Nonetheless, there is a gap in knowledge regarding clinical trials focusing solely on HFmrEF patient subgroups [35,36]. 

C-reactive protein (CRP) serves as a conventional marker of systemic inflammation; it is primarily produced by hepatocytes and cardiovascular tissue in response to infection, cell invasion, or tissue injury. High-sensitivity CRP can detect mild inflammation at much lower concentrations than conventional CRP. A post-hoc analysis of 843 patients in the PROSPECT study showed no significant differences in CRP levels among patients with the three types of chronic HF. With the introduction of HFmrEF, additional biomarkers have been utilised to assess this condition. However, it is noteworthy that the latest ESC guideline in 2021 only mentions natriuretic peptides as recommended biomarkers for evaluation [37,38]. 

## 5. Management

Understanding the fundamental pathophysiological mechanisms has the potential to improve patient outcomes and also helps with the precise targeting of treatment options. Management begins with the risk stratification of patients, followed by pharmacological and non-pharmacological therapies. 

### 5.1. Risk Stratification Model

**Figure 3:**
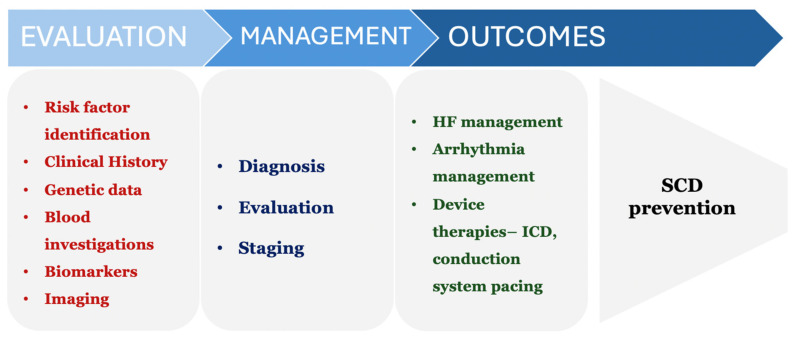
Risk stratification model to evaluate and monitor Heart failure with mildly reduced ejection fraction patients. Abbreviations: SCD, sudden cardiac death; ICD, intracardiac defibrillator.

A comprehensive risk stratification model (Figure 3) for patients exhibiting HF with mildly reduced ejection fraction (HFmrEF) can significantly improve patient outcomes by identifying high-risk individuals early and tailoring their management accordingly. An outline of such a model is presented in the following subsections.

#### 5.1.1. Initial Patient Evaluation and Risk Factor Assessment

- Conduct a thorough evaluation of the patient’s medical history, including cardiovascular risk factors such as hypertension, diabetes, obesity, smoking, and a family history of cardiovascular diseases.
- Perform genetic testing to identify inherited cardiomyopathies, which can provide insights into the underlying etiology and guide treatment decisions.
- Utilise clinical investigations, including biomarkers such as B-type natriuretic peptide (BNP) and troponin, to assess myocardial injury and predict cardiac dysfunction.
- Employ cardiac imaging modalities such as echocardiography and CMR to evaluate ventricular function, detect myocardial scarring due to fibrosis, and assess the overall cardiac structure and function.

#### 5.1.2. Diagnosis and Staging

- Diagnose HFmrEF based on clinical criteria, including symptoms of HF (e.g., dyspnea, fatigue, edema) and objective evidence of cardiac dysfunction with an LVEF of between 40% and 49%.
- Stage the patients based on disease severity, using established criteria such as the New York Heart Association (NYHA) functional classification and the ACC/AHA staging system, taking into account their symptoms, functional limitations, and objective evidence of structural heart disease.

#### 5.1.3. Heart Failure Management

- The treatment approach recommended by the guidelines encompasses the utilisation of angiotensin-converting enzyme inhibitors (ACEIs), angiotensin receptor blockers (ARBs), mineralocorticoid receptor antagonists (MRAs), sacubitril/valsartan (ARNIs), beta-blockers, and sodium-glucose cotransporter 2 inhibitors (SGLT2i).
- Offer thorough patient education and counselling regarding lifestyle adjustments, which may involve dietary alterations, participation in exercise regimens, cessation of smoking, and management of weight.

#### 5.1.4. Arrhythmia Management and Device Therapies

- Evaluate patients for arrhythmias, including atrial fibrillation and ventricular arrhythmias, and initiate appropriate management strategies such as rate or rhythm control and anticoagulation therapy [39].
- Consider implantable cardioverter-defibrillator (ICD) therapy for the primary prevention of sudden cardiac death in high-risk patients, who are selected based on a history of sustained ventricular arrhythmias [40].

#### 5.1.5. Risk Prediction and Long-Term Outcomes

- Integrate clinical, genetic, biomarker, and imaging data to develop a risk prediction model for identifying patients at high risk of adverse outcomes such as sudden cardiac death and poor long-term prognosis.
- Use validated risk scores or machine learning algorithms to stratify patients into different risk categories and tailor their treatment strategies accordingly, focusing on intensive surveillance and targeted interventions for high-risk individuals.
- Implement a multidisciplinary approach involving cardiologists, electrophysiologists, HF specialists, genetic counsellors, and other healthcare providers to optimise patient care and improve outcomes over the long term.

By incorporating these components into a comprehensive risk stratification model, clinicians can better identify and manage HFmrEF patients, which ultimately leads to improved clinical outcomes and quality of life.

### 5.2. Pharmacological Treatment

The 2021 ESC guidelines, advocate for a comprehensive pharmacological approach in managing HFmrEF, recommending angiotensin-converting enzyme inhibitors (ACEIs), angiotensin receptor blockers (ARBs) [41], mineralocorticoid receptor antagonists (MRAs) [42], sacubitril/valsartan (ARNIs) [43,44], beta-blockers [45], and sodium-glucose cotransporter 2 inhibitors (SGLT2) [46,47,48,49,50,51] as Class 2b recommendations (Table 1).

Candesartan, as per post-hoc analyses of CHARM data [41], demonstrated significant reductions in cardiovascular events and HF hospitalisations, particularly in patients with recurrent HFmrEF episodes. Similarly, the secondary analysis of the TOPCAT trial [42] revealed decreased mortality rates with MRAs among individuals with an EF ranging from 44 to 49%. However, the findings from a prespecified combined analysis of PARADIGM-HF and PARAGON-HF trials [43,44] suggest limited benefits of ARNIs in HFmrEF patients with an EF of 40–50%, although the data indicate potential advantages, especially in women with mild decreases in EF. Moreover, meta-analysis [45] data highlight the efficacy of beta blockers in reducing cardiovascular mortality by half in HFmrEF patients with sinus rhythm. Sub-analysis of the DIG trial demonstrated that digoxin did not improve clinical outcomes in HFmrEF. Importantly, the trial only comprised patients in sinus rhythm [46].

Additionally, SGLT2 inhibitors have shown promising results in reducing cardiovascular death or HF hospitalisation, as demonstrated in the EMPEROR-preserved study and the DELIVER trial [47,48,49,50,51], especially in patients with mildly reduced or preserved EF, irrespective of their diabetic status. Lastly, vericiguat has demonstrated its efficacy in reducing the combined primary endpoint of cardiovascular death or first HF hospitalisation, albeit less so in patients with an EF percentage between 40 and 45% [52]. These findings underscore the evolving landscape of pharmacotherapy for HFmrEF, emphasising the need for tailored treatment strategies to optimise patient outcomes.

### 5.3. Non-Pharmacological Treatment and Comorbidities Management

#### 5.3.1. Pacing in Heart Failure with Mildly Reduced Ejection Fraction 

According to the 2023 guidelines on cardiac physiological pacing, endorsed by HRS/APHRS/LAHRS, to prevent and alleviate heart failure, there is a recommendation (Class 2a) for the use of cardiac resynchronisation therapy (CRT/His bundle pacing/left bundle area pacing) in heart failure patients with a left ventricular ejection fraction (LVEF) falling between 36% and 50%, in the event that they are expected to require significant ventricular pacing exceeding 40%. Additionally, there is a Class 2b indication for HFmrEF patients with left bundle branch block (LBBB), a QRS duration greater than 150 milliseconds, and New York Heart Association (NYHA) classes II–IV symptoms. Pacing is also recommended in HFmrEF patients with atrioventricular (AV) block and patients with atrial fibrillation (AF) who are undergoing AV node ablation and have a QRS duration of less than 120 milliseconds (class IIa) [53].

**Figure 4:**
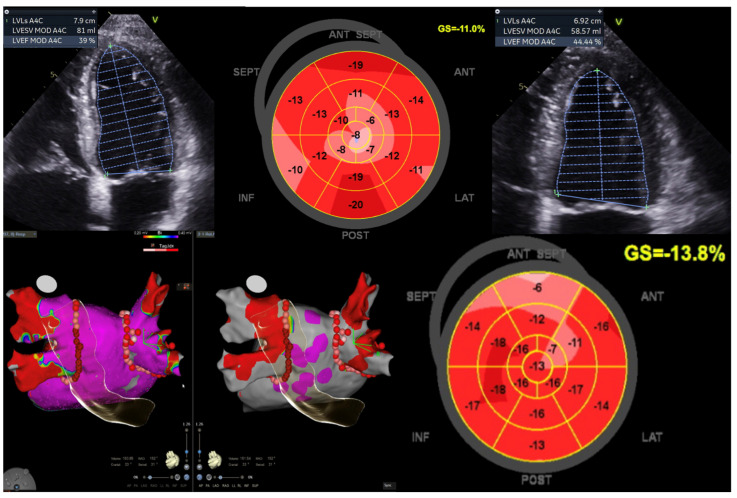
Illustration of LV function improvement (EF 44.4%, GLS −13.8%) in a 52-year-old male diagnosed with Tachy-Brady syndrome with LV dysfunction (EF 39%, GLS −11%), who underwent dual chamber pacemaker-left bundle branch area pacing, followed by atrial fibrillation ablation (pulmonary vein isolation). This case underscores the significance of conduction system pacing and echocardiography when using a global longitudinal strain (GLS) assessment for evaluating improvement in LV function. Image sourced from the Canberra Heart Rhythm Centre.

In traditional pacing protocols, initiating the electrical impulse from the right ventricle can disrupt myocardial coordination, resulting in compromised cardiac output efficiency. Conversely, contemporary methods such as left bundle branch area pacing (LBBAP) or His bundle pacing (HBP) offer a more targeted approach by directly stimulating the heart’s intrinsic conduction pathways. In cases of HFmrEF, delays in ventricular activation contribute to exacerbated HF symptoms (Figure 4). Through conduction system pacing (CSP), electrical depolarisation is delivered directly to the ventricular myocardium, minimising temporal disparities and promoting optimal electromechanical synchrony. This intervention holds some potential for enhancing cardiac performance in affected individuals.

By pacing the LBB, the electrical activation of the heart can be more physiological, allowing for better synchronisation of the ventricular contractions thereby improving the overall LV function and improving the symptoms associated with LV dysfunction. Shroff, J.P., et al. studied the role of CSP in 101 HF patients referred for cardiac resynchronisation therapy (CRT). They found that the LBBAP-CRT group showed greater improvement in LVEF at 6 and 12 months, accompanied by a greater reduction in LV end-systolic volume. This suggests that the use of LBBAP-CRT results in a meaningful improvement in quality of life (QoL) and a reduction in HF hospitalisations [54]. 

The anatomical characteristics of the left bundle branch (LBB) determine the feasibility of LBBAP as a potential physiological pacing approach. Unlike HBP, LBBAP, which relies on capturing the LBB and distal conduction system tissues, offers a broader target area for pacing that may extend beyond the location of the block in the distal HB. In contrast, with pacing at the right ventricular apex, HBP does not induce interventricular or intraventricular asynchrony and does not trigger myocardial perfusion abnormalities. 

Identifying the conduction system’s location can be challenging; fluoroscopy may not be enough. Enhancing CSP outcomes relies heavily on cardiac imaging. The CHIPS study aimed to evaluate lead locations using cardiac computerised tomography (CT) in 100 consecutive patients undergoing physiological pacing. The results showed that LBB pacing exhibited versatility in the septal regions and allowed for selective fascicle capture, with deeper lead penetration. The study emphasises the role of additional imaging in guiding lead depth for effective pacing [55].

#### 5.3.2. Intra-Cardiac Defibrillator Therapy

Limited data exist regarding the risk of SCD and the need for intra-cardiac defibrillator (ICD) therapy in HFmrEF. Raja D.C et al. [56] studied the demographic, clinical, device therapy, and survival characteristics of mixed cardiomyopathy (CMP) in a cohort of patients implanted with a defibrillator. They found that the trends of long-term prognosis of patients with mixed CMP are worse than in non-ischemic cardiomyopathy and similar to those in ischemic cardiomyopathy. Patients with an LVEF percentage between 40 and 50% and with specific forms of cardiomyopathy, such as those due to genetic mutations (e.g., Lamin A/C, filamin-C, and phospholamban), arrhythmogenic LV CMP, or hypertrophic CMP, may require ICD therapy. In such cases, the decision for ICD implantation relies on specific risk factors for SCD rather than solely relying on the LVEF percentage being <35%. 

#### 5.3.3. Inter-Atrial Shunt Devices

Given that elevated left atrial pressure is one of the pathophysiological mechanisms of HF, attention has turned toward the development of interatrial shunt devices or unidirectional left-right shunting. Studies involving patients with an LVEF percentage of >40% and elevated left-sided filling pressures have shown that these devices are linked with improved hemodynamics (Table 2), although they have not demonstrated a reduction in HF events or enhancement in health status among HF patients with an LVEF percentage of >40% [57,58,59].

### 5.4. Treatment of Arrhythmias 

#### 5.4.1. Atrial Fibrillation Ablation

Atrial fibrillation (AF), the most common arrhythmia, heightens the mortality and hospitalisation risks in HF patients, with HF-related structural and neurohormonal changes that exacerbate AF onset and progression. Post hoc analysis of the CABANA trial results showed that catheter ablation as a rhythm control strategy was not shown to improve survival compared to antiarrhythmic drugs in patients with LVEF percentages of 40–50% (Table 2) [60]. The small sample size might limit interpretation of the results.

**Figure 5:**
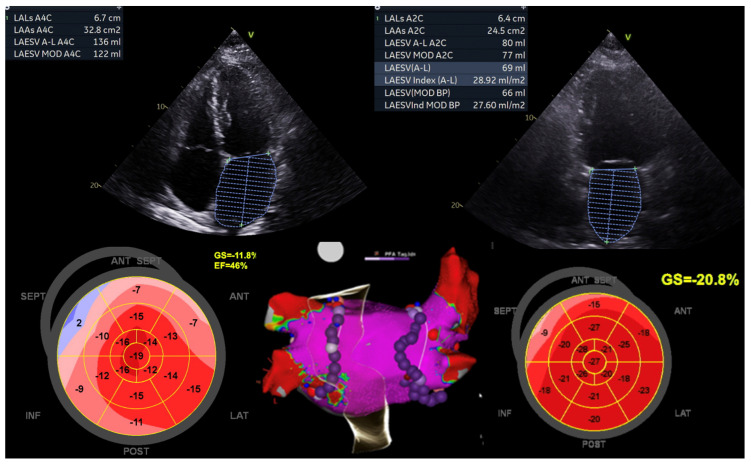
This image emphasises the positive impact of atrial fibrillation (AF) ablation on enhancing outcomes and quality of life for a 64-year-old woman with persistent AF and HFmrEF. Initially, she had a baseline left ventricular ejection fraction (LVEF) of 46%, a left atrial area (LAA) of 32.8 cm^2^, and a 100% AF burden. Following AF ablation (pulmonary vein isolation), her LVEF percentage significantly increased to 68%, and her LAA reduced to 24.5 cm^2^ within 3 months, accompanied by an improvement in global longitudinal strain (GLS) from −11.8% to −20.8%. Subsequent Holter monitoring showed no AF recurrence. Image courtesy of the Canberra Heart Rhythm Centre.

AF ablation significantly enhances LVEF, irrespective of LV dysfunction severity (Figure 5). In select AF patients with HF and uncontrolled heart rate (HR), a pace and ablate strategy can improve LVEF and functional class. This approach, however, carries the risk of ventricular dyssynchrony and SCD post-atrioventricular (AV) node ablation. To avert mechanical dyssynchrony and HF exacerbation, CSP offers a viable alternative [64]. Moreover, elevated pulmonary artery systolic pressure, increased serum creatinine, and reduced baseline LVEF independently predict the composite endpoint of all-cause mortality or HF hospitalisation. 

#### 5.4.2. Ventricular Arrhythmia Ablation

**Figure 6:**
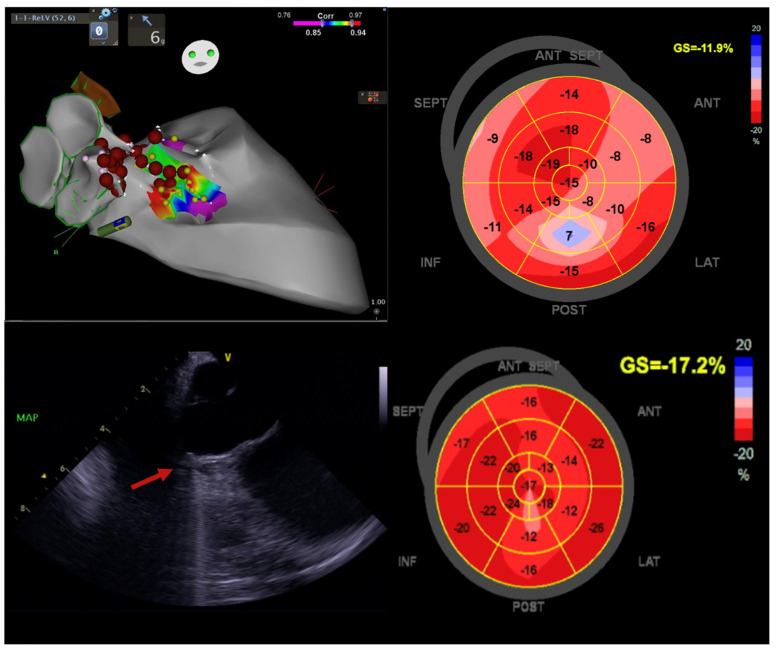
Image emphasising the significance of PVC ablation in a 60-year-old male diagnosed with HFmrEF. Initially, his left ventricular ejection fraction (LVEF) percentage stood at 42%, and his global longitudinal strain (GLS) measured −11.9%. The baseline PVC burden was noted at 22%, with PVCs localised to the right para-Hisian region. Intracardiac echocardiography unveiled a basal septal scar (indicated by a red arrow) positioned just below the right coronary cusp (RCC). At the one-year follow-up, the PVC burden plummeted to 1.2%, accompanied by an improvement in LVEF to 66% and GLS to 17.2%. This case highlights the pivotal role of PVC ablation in ameliorating left ventricular function and enhancing quality of life. Image courtesy of the Canberra Heart Rhythm Centre.

Ventricular arrhythmias (VAs) are a common type of rhythm disorder characterised by extra heartbeats originating in the ventricles rather than the sinoatrial node. They can be asymptomatic or can present as palpitations, dizziness, exercise intolerance, or sudden cardiac death. While occasional premature ventricular complexes (PVCs) are usually harmless, frequent PVCs or those causing symptoms may require treatment. One treatment option for managing frequent PVCs is ablation therapy. Before proceeding with PVC ablation, a thorough evaluation is necessary. (Figure 6) This includes a detailed medical history, a physical examination, an electrocardiogram, an echocardiogram, and possibly ambulatory monitoring to assess the PVC burden. Using an electrophysiological study (EPS), the source of PVCs can be mapped within the heart and catheters are positioned at the site(s) responsible for generating PVCs. Energy, usually in the form of radiofrequency ablation treatment or cryotherapy, is delivered to the target tissue to disrupt the abnormal electrical pathways [65].

### 5.5. Management of Ischemia in Heart Failure with Mildly Reduced Ejection Fraction

Post hoc analyses conducted on the ISCHEMIA trial results revealed that an invasive approach yielded superior outcomes in reducing CV death or myocardial infarction when compared to a conservative strategy among patients with at least moderate ischemia and an LVEF percentage between 35 and 45% (Table 2) [61].

### 5.6. Exercise

In the HART trial, physical inactivity was associated with almost double the all-cause and cardiovascular mortality in HF patients with NYHA II/III and with preserved or reduced LVEF, whereas even modest exercise was linked to improved survival [63] (Table 2).

## 6. Current State of Heart Failure with Mildly Reduced Ejection Fraction

**Figure 7:**
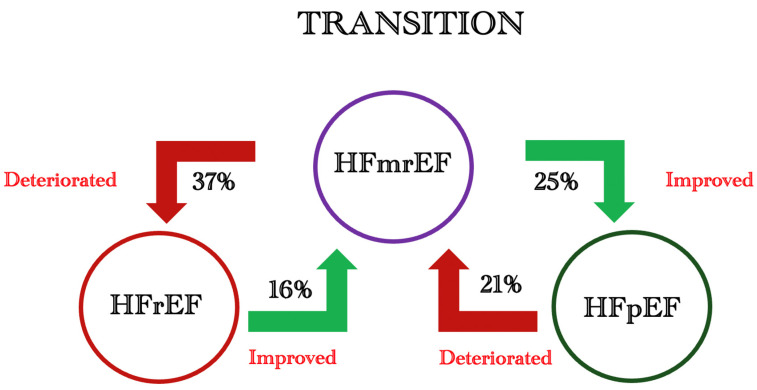
Graphic illustrating LV function trends among HF patients.

The transition of patients from HFpEF to HFmrEF and their further deterioration from HFmrEF to HFrEF represents a critical challenge in the management of heart failure (Figure 7). Seminal studies involving 4942 patients have shed light on this complex dynamic, revealing concerning trends [66]. Among HFmrEF patients, a notable 37% deteriorated to HFrEF, while only 16% showed improvement from HFrEF to HFmrEF. Conversely, HFpEF patients demonstrated a lower but still significant tendency toward transition, with 21% deteriorating to HFmrEF and 25% improving to a preserved ejection fraction (Figure 7). This data underscores the heightened risk that HFmrEF patients face in progressing to HFrEF, highlighting the need for vigilant monitoring and proactive management strategies to mitigate such transitions and optimise patient outcomes.

There are several notable challenges that impact the management and outcomes of affected individuals. One significant issue is the absence of standardised risk stratification models tailored specifically for HFmrEF patients, leading to difficulties in accurately identifying and evaluating their condition. There needs to be a comprehensive risk assessment model to help clinicians effectively risk-stratify patients based on their likelihood of adverse events or disease progression. This will lead to optimal treatment decisions and outcomes.

Furthermore, there is a noticeable lack of clear guidelines regarding the indications for catheter ablations for tachyarrhythmias and device-based therapies aimed at preventing SCD to improve overall prognosis in HFmrEF patients. While these interventions have demonstrated efficacy in certain subsets of HF patients, their utility and optimal application in the context of HFmrEF remain uncertain, due to limited evidence and guidance. This ambiguity complicates clinical decision-making and may contribute to the variability of physician care.

Additionally, the transition of patients from HFpEF to HFmrEF and their subsequent deterioration from HFmrEF to HFrEF poses a critical challenge in HF management. This dynamic progression underscores the heterogeneity and complexity of HFmrEF, as well as the need for nuanced and individualised approaches to patient care. Addressing these challenges requires interdisciplinary collaboration, ongoing research efforts, and the development of evidence-based guidelines tailored specifically for HFmrEF, ultimately aiming to improve outcomes and quality of life for affected individuals.

## 7. Chronic Kidney Disease and Heart Failure with Mildly Reduced Ejection Fraction

Chronic kidney disease (CKD) is prevalent in HF and is linked to poorer outcomes. The intricate interaction between the heart and kidneys, known as the cardiorenal syndrome, plays a role. Higher mortality rates with CKD and declining renal function are well-documented in HFrEF. However, the connection between CKD and HfmrEF, as well as HFpEF, remains unclear.

Löfman et al. analyzed data from the Swedish Heart Failure Registry, categorising patients based on EF levels. They found that CKD prevalence was highest in HFpEF patients, followed by HFmrEF and HFrEF patients. Despite similar associations between covariates and CKD across EF groups, CKD was more strongly associated with mortality in HFrEF and HFmrEF patients compared to HFpEF. Furthermore, CKD was a better predictor of death in HFrEF and HFmrEF patients compared to HFpEF. These findings suggest that while CKD plays a significant role in all types of HF, its impact on mortality and prognostic discrimination varies across EF categories.

While this study enhances our understanding of CKD in various HF types, the underlying mechanisms remain speculative. In HFrEF, the cardiorenal syndrome is well documented, likely capturing patients with advanced HF. CKD in this context results from both backward and forward failure, sympathetic activation, and neurohormonal changes, worsening cardiac function. In HFmrEF and HFpEF, the link is less clear. CKD and HFmrEF may reflect age and comorbidities, evolve independently, or stem from similar risk factors. Increased central venous pressure correlates with impaired renal function in reduced, mildly reduced, and preserved EF patients.

Lower renin-angiotensin-aldosterone system (RAAS) inhibitor use in patients with declining renal function is expected but raises concerns. Observational data suggest that using RAAS inhibitors might benefit even patients with severe renal failure. Understanding the impact of RAAS inhibitor use on outcomes in HFpEF and HFmrEF, especially with CKD, remains crucial for future trial design [67].

## 8. Prognosis

**Figure 8:**
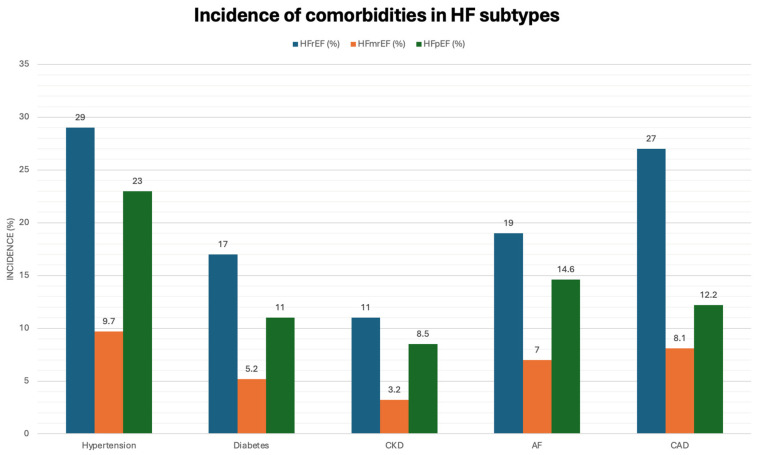
Highlights the incidence of comorbidities in various subtypes of heart failure. It is interesting to note the lower incidence of comorbid conditions in HFmrEF, highlighting possible independent risk factors involved in the prognosis of these patients [66]. Identifying these could be the first step in predicting HFmrEF patients progressing to HFrEF.

Long-term all-cause mortality risk is initially lower in HFmrEF patients compared to HFrEF patients at 1, 2, and 3 years, but becomes insignificant thereafter. However, mortality risk is similar between HFmrEF and HFpEF patients. Beyond 3 years, differences in mortality risk between HFmrEF and HFrEF tend to even out, possibly due to transitions in LVEF over time or insufficient statistical power in very long-term studies. Patients transitioning between HFmrEF, HFrEF, and HFpEF categories over time indicates the heterogeneous nature of HFmrEF. LVEF alone may not accurately predict prognosis in HFmrEF, emphasising the importance of additional prognostic indicators such as associated comorbidities (Figure 8), cardiac MRI, and arrhythmia burden [66].

RCTs exhibit larger disparities across EF groups, with HFpEF and HFmrEF patients experiencing lower cardiovascular event risks compared to HFrEF patients. However, RCTs typically include younger, healthier patients with more severe HF. In the CHARM program, HFmrEF patients had significantly lower all-cause death rates than was suggested by registry estimates. A steep decrease in event rates with increasing EF was observed until specific thresholds were reached, after which the curves flattened. Non-cardiovascular adverse outcomes were generally higher in HFpEF. Nonetheless, the TIME-CHF trial found similar hospital admission rates and mortality regardless of EF [68].

## 9. Future Directions

The accumulated insights gained from numerous previous studies have brought forth myriad intriguing questions pertaining to the precise phenotyping of HFmrEF and to devising more targeted treatment strategies. However, despite these efforts, there remain significant uncertainties regarding the most effective management of HFmrEF, which may leave healthcare practitioners facing a dilemma. Primarily, the majority of HFmrEF treatment data stem primarily from trials based on the conventional classification of HF groups, which rely on LVEF. However, a recent understanding of HF pathophysiology has shed light on the limitations of using LVEF categories to define individual patient phenotypes.

Large-scale studies are essential to unravel which clinical features best predict outcomes and responses to treatment. Moreover, there is growing optimism surrounding the potential use of biomarker-based phenotyping approaches in HFmrEF, which may aid in identifying new potential treatment targets. Clinicians may gain deeper insights into the pathophysiological mechanisms at play in HFmrEF, thus making way for a more tailored and efficacious treatment strategy.

## 10. Conclusions

Recognising HFmrEF as a clinical syndrome characterised by various contributing risk factors, comorbidities, phenotypic manifestations, disease duration, and natural history is crucial. Consequently, individualised management strategies tailored to each patient, considering a combination of HF symptoms and comorbidities, will be essential.

HFmrEF continues to attract attention due to its overlapping features with both HFrEF and HFpEF across different populations. While multiple drug therapies have demonstrated efficacy in mitigating adverse clinical outcomes in HFpEF, most of the benefits seem to be concentrated in patients with either mildly reduced or preserved EF at the lower end of the spectrum. There is increasing evidence supporting the effectiveness of neurohormonal antagonism in HFmrEF, suggesting that it may represent an extension of the HFrEF spectrum, which has traditionally been excluded from HFrEF trials. This raises the question of whether HFmrEF will be treated as a distinct entity with dedicated trials or potentially be integrated into HFrEF trials for patients with LVEF percentages of <40% in the future.

## Figures and Tables

**Table 1:** Summary of pharmacological studies involving HFmrEF patients. Abbreviations: RCT, randomised control trial; HF, heart failure; CV, cardiovascular.

THERAPY	AUTHORS	TYPE OF STUDY	NO. OF PATIENTS	LVEF	FOLLOW UP	PRIMARY EVENTS/100 PATIENT YEARS	OUTCOMES
**Candesartan (ARB)**	Lund et al., 2018 [41]	Post hoc analysis of CHARM	1322	40–49%	2.9 years	significant reduction vs placebo: 0.76 (0.61–0.96)	HF hospitalizations: 0.72 (0.55–0.95) CV death: 0.81 (0.60–1.11) All cause death: 0.79 (0.60–1.04)
**Spironolactone (MRA)**	Solomon et al., 2020 [42]	Post hoc analysis of TOPCAT	520	44–49%	3.1 years	no significant reduction vs placebo: 0.89 (0.77–1.04)	HF hospitalization: 0.76(0.46–1.27) CV death: 0.69(0.43–1.12) All cause death: 0.73 (0.49–1.10)
**Sacubitril/Valsartan (ARNI)**	Solomon et al., 2019 [44]	Prespecified pooled analysis of PARADIGM-HF and PARAGON-HF	730	40–50%	2.3 years	no significant reduction vs ACE(-): 0.94 (0.69–1.28)	HF hospitalization: 0.81 (0.59–1.13) CV death: 0.98 (0.66–1.46)
**Beta Blockers**	Cleland et al. [45]	Metanalysis of 11 RCT’s	575	40–49%			CV hospitalisation: 0.95 (0.68–1.32) CV death: 0.48 (0.24–0.97) all cause death: 0.59 (0.34–1.03)
**Digoxin**	Abful-Rahim et al., 2018 [46]	Post hoc analysis of DIG trial	1195	40–49%	3.1 years	no significant reduction vs. placebo: 0.83 (0.66–1.05)	HF hospitalizations: 0.80 (0.63–1.03) CV death: 1.24 (0.94–1.61) all cause death: 1.08 (0.85–1.37)
**Empagliflozin**	Anker et al., 2021 [47]	Subgroup analysis of EMPEROR-Preserved	1983	41–49%	2.2 years	significant reduction vs. placebo: 0.79 (0.69–0.90)	CV death or HF hospitalizations: 0.71 (0.57–0.88)
**Vericiguat**	Armstrong PW et al., 2020 [52]	Subgroup analysis of VICTORIA trial	236	41–45%	10.8 months	no significant reduction vs placebo: 0.90 (0.82–0.98)	CV death or HF hospitalization: 1.05 (0.81–1.36)

**Table 2:** Summary of non-pharmacological studies involving HFmrEF patients. Abbreviations: AF, atrial fibrillation; HF, heart failure; CV, cardiovascular; MI, myocardial infarction.

THERAPY	AUTHORS	TYPE OF STUDY	NO. OF PATIENTS	LVEF	FOLLOW UP	PRIMARY EVENTS/100 PATIENT YEARS	OUTCOMES
**Interatrial shunt device**	Shah et al., 2018 [59]	REDUCE LAP HF	44	>40%	1 year	no difference vs placebo: 0.63 (0.33–1.21)	CV death, stroke, HD events: 1.0 (0.8–1.2)
**AF ablation**	Packer et al., 2019 [60]	post hoc analysis of CABANA trial	67	40–49%	5 year	significant reduction, ablation vs drug therapy: 0.64 (0.41–0.99)	All cause death: 0.85 (0.22–3.22)
**revascularisation**	Lopes et al., 2020 [61]	post hoc analysis of ISCHEMIA trial	221	35–45%	3.2 years	significant reductions invasive vs conservative (event rate): −12.1% [−22.6, −1.6%]	CV death, MI, resuscitated cardiac arrest, hospitalisation for HF: 0.62 (0.40–0.97)
**Iron supplements**	Ponikowski et al., 2015 [62]	AFFIRM AHF	1132	<50%	1.1years	significant reductions vs placebo: 0.39 (0.19–0.82)	HF hospitalisations: 0.74 (0.58–0.94) CV death: 0.94 (0.70–1.32)
**Exercise**	Doukky et al., 2017 [63]	Sub analysis of HART trial	902	all HF groups	3 years	significant reduction of moderate exercise vs inactivity: 1.65 (1.10–2.48)	All cause death: 2.01 (2.47–3.00) Cardiac death: 2.01 (1.28–3.17)

## References

[1] Coronel R., de Groot J.R., van Lieshout J.J. (2001). Defining heart failure. Cardiovasc. Res..

[2] Tan L.B., Williams S.G., Tan D.K., Cohen-Solal A. (2010). So many definitions of heart failure: Are they all universally valid? A critical appraisal. Expert. Rev. Cardiovasc. Ther..

[3] Ponikowski P., Voors A.A., Anker S.D., Bueno H., Cleland J.G., Coats A.J., Falk V., González-Juanatey J.R., Harjola V.P., Jankowska E.A. (2016). Authors/Task Force Members. 2016 ESC guidelines for the diagnosis and treatment of acute and chronic heart failure: The Task Force for the diagnosis and treatment of acute and chronic heart failure of the European Society of Cardiology (ESC) Developed with the special contribution of the Heart Failure Association (HFA) of the ESC. Eur. Heart J..

[4] Ponikowski P., Anker S.D., AlHabib K.F., Cowie M.R., Force T.L., Hu S., Jaarsma T., Krum H., Rastogi V., Rohde L.E. (2014). Heart failure: Preventing disease and death worldwide. ESC Heart Fail..

[5] Mozaffarian D., Benjamin E.J., Go A.S., Cushman M., Das S.R., Deo R., de Ferranti S.D., Floyd J., Fornage M., Gillespie C. (2016). American Heart Association Statistics Committee; Stroke Statistics Subcommittee. Heart Disease and Stroke Statistics-2016 Update: A report from the American Heart Association. Circulation.

[6] Crespo-Leiro M.G., Metra M., Lund L.H., Milicic D., Costanzo M.R., Filippatos G., Gustafsson F., Tsui S., Barge-Caballero E., De Jonge N. (2018). Advanced heart failure: A position statement of the Heart Failure Association of the European Society of Cardiology. Eur. J. Heart Fail..

[7] Ahmad T., Lund L.H., Rao P., Ghosh R., Warier P., Vaccaro B., Dahlström U., O’Connor C.M., Felker G.M., Desai N.R. (2018). Machine learning methods improve prognostication, identify clinically distinct phenotypes, and detect heterogeneity in response to therapy in a large cohort of heart failure patients. J. Am. Heart Assoc..

[8] Lund L.H. (2016). Heart failure with “mid-range” ejection fraction—New opportunities. J. Card. Fail..

[9] Lund L.H., Vedin O., Savarese G. (2018). Is ejection fraction in heart failure a limitation or an opportunity? Eur. J. Heart Fail..

[10] Lam C.S., Solomon S.D. (2014). The middle child in heart failure: Heart failure with mid-range ejection fraction (40–50%) Eur. J. Heart Fail..

[11] Bozkurt B., Coats A.J.S., Tsutsui H., Abdelhamid C.M., Adamopoulos S., Albert N., Anker S.D., Atherton J., Böhm M., Butler J. (2021). Universal definition, and classification of heart failure: A report of the Heart Failure Society of America, Heart Failure Association of the European Society of Cardiology, Japanese Heart Failure Society and Writing Committee of the Universal Definition of Heart Failure: Endorsed by Canadian Heart Failure Society, Heart Failure Association of India, the Cardiac Society of Australia and New Zealand, and the Chinese Heart Failure Association. Eur. J. Heart Fail..

[12] Lam C.S.P., Voors A.A., Piotr P., McMurray J.J.V., Solomon S.D. (2020). Time to rename the middle child of heart failure: Heart failure with mildly reduced ejection fraction. Eur. Heart J..

[13] McMurray J.J.V., Adamopoulos S., Anker S.D., Auricchio A., Böhm M., Dickstein K., Falk V., Filippatos G., Fonseca C., Gomez-Sanchez M.A. (2012). ESC Guidelines for the diagnosis and treatment of acute and chronic heart failure 2012: The Task Force for the Diagnosis and Treatment of Acute and Chronic Heart Failure 2012 of the European Society of Cardiology. Developed in collaboration with the Heart. Eur. J. Heart Fail..

[14] Savarese G., Lund L.H. (2017). Global public health burden of heart failure. Card. Fail. Rev..

[15] Groenewegen A., Rutten F.H., Mosterd A., Hoes A.W. (2020). Epidemiology of heart failure. Eur. J. Heart Fail..

[16] Hunt S.A., Abraham W.T., Chin M.H., Feldman A.M., Francis G.S., Ganiats T.G., Jessup M., Konstam M.A., Mancini D.M., Michl K. (2005). ACC/AHA 2005 Guideline Update for the Diagnosis and Management of Chronic Heart Failure in the Adult: A report of the American College of Cardiology/American Heart Association Task Force on Practice Guidelines (Writing Committee to Update the 2001 Guidelines for the Evaluation and Management of Heart Failure): Developed in collaboration with the American College of Chest Physicians and the International Society for Heart and Lung Transplantation: Endorsed by the Heart Rhythm Society. Circulation.

[17] Sweitzer N.K., Lopatin M., Yancy C.W., Mills R.M., Stevenson L.W. (2008). Comparison of clinical features and outcomes of patients hospitalized with heart failure and normal ejection fraction (>or =55%) versus those with mildly reduced (40% to 55%) and moderately to severely reduced (<40%) fractions. Am. J. Cardiol..

[18] Stolfo D., Uijl A., Vedin O., Strömberg A., Faxén U.L., Rosano G.M., Sinagra G., Dahlström U., Savarese G. (2019). Sex-Based Differences in Heart Failure Across the Ejection Fraction Spectrum: Phenotyping, and Prognostic and Therapeutic Implications. JACC Heart Fail..

[19] Fonarow G.C., Stough W.G., Abraham W.T., Albert N.M., Gheorghiade M., Greenberg B.H., O’Connor C.M., Sun J.L., Yancy C.W., Young J.B. (2007). Characteristics, treatments, and outcomes of patients with preserved systolic function hospitalized for heart failure: A report from the OPTIMIZE-HF registry. J. Am. Coll. Cardiol..

[20] Tromp J., Westenbrink B.D., Ouwerkerk W., van Veldhuisen D.J., Samani N.J., Ponikowski P., Metra M., Anker S.D., Cleland J.G., Dickstein K. (2018). Identifying pathophysiological mechanisms in heart failure with reduced versus preserved ejection fraction. J. Am. Coll. Cardiol..

[21] Rastogi A., Novak E., Platts A.E., Mann D.L. (2017). Epidemiology, pathophysiology and clinical outcomes for heart failure patients with a mid-range ejection fraction. Eur. J. Heart Fail..

[22] Savarese G., Vedin O., D’Amario D., Uijl A., Dahlström U., Rosano G., Lam C.S., Lund L.H. (2019). Prevalence and Prognostic Implications of Longitudinal Ejection Fraction Change in Heart Failure. JACC Heart Fail..

[23] Lupón J., Gavidia-Bovadilla G., Ferrer E., de Antonio M., Perera-Lluna A., López-Ayerbe J., Domingo M., Núñez J., Zamora E., Moliner P. (2018). Dynamic Trajectories of Left Ventricular Ejection Fraction in Heart Failure. J. Am. Coll. Cardiol..

[24] Solomon S.D., Anavekar N., Skali H., McMurray J.J., Swedberg K., Yusuf S., Granger C.B., Michelson E.L., Wang D., Pocock S. (2005). Influence of Ejection Fraction on Cardiovascular Outcomes in a Broad Spectrum of Heart Failure Patients. Circulation.

[25] Chang W., Lin C.H., Hong C., Liao C.-T., Liu Y.-W., Chen Z.-C., Shih J.-Y. (2021). The predictive value of global longitudinal strain in patients with heart failure mid-range ejection fraction. J. Cardiol..

[26] Kalam K., Otahal P., Marwick T.H. (2014). Prognostic implications of global LV dysfunction: A systematic review and meta-analysis of global longitudinal strain and ejection fraction. Heart.

[27] Stanton T., Leano R., Marwick T.H. (2009). Prediction of all-cause Mortality from Global Longitudinal Speckle Strain. Circ. Cardiovasc. Imaging.

[28] Rommel K., Lücke C., Lurz P. (2017). Diagnostic and Prognostic Value of CMR T 1 -Mapping in Patients with Heart Failure and Preserved Ejection Fraction. Rev. EspanñOla De. Cardiol..

[29] Raja D.C., Samarawickrema I., Srinivasan J.R., Menon S., Das S.K., Jain S., Tuan L.Q., Desjardins B., Marchlinski F.E., Abhayaratna W.P. (2023). Correlation of myocardial strain by CMR-feature tracking with substrate abnormalities detected by electro-anatomical mapping in patients with nonischemic cardiomyopathy. J. Interv. Card. Electrophysiol..

[30] Savarese G., Musella F., D’Amore C., Vassallo E., Losco T., Cecere M., Petraglia L., Trimarco B., Perrone-Filardi P. (2014). Changes of Natriuretic Peptides Predict Hospital Admissions in Patients with Chronic Heart Failure: A meta- analysis. JACC Heart Fail..

[31] Anwaruddin S., Lloyd-Jones D.M., Baggish A., Chen A., Krauser D., Tung R., Chae C., Januzzi J.L. (2006). Renal function, congestive heart failure, and amino-terminal pro-brain natriuretic peptide measurement: Results from the ProBNP Investigation of Dyspnea in the Emergency Department (PRIDE) Study. J. Am. Coll. Cardiol..

[32] Kociol R.D., Pang P.S., Gheorghiade M., Fonarow G.C., O’Connor C.M., Felker G.M. (2010). Troponin elevation in heart failure prevalence, mechanisms, and clinical implications. J. Am. Coll. Cardiol..

[33] Latini R., Masson S., Anand I.S., Missov E., Carlson M., Vago T., Angelici L., Barlera S., Parrinello G., Maggioni A.P. (2007). Prognostic Value of Very Low Plasma Concentrations of Troponin T in Patients with Stable Chronic Heart Failure. Circulation.

[34] Yu L., Ruifrok W.P.T., Meissner M., Bos E.M., van Goor H., Sanjabi B., van der Harst P., Pitt B., Goldstein I.J., Koerts J.A. (2013). Genetic and Pharmacological Inhibition of Galectin- 3 Prevents Cardiac Remodelling by Interfering with Myocardial Fibrogenesis. Circ. Heart Fail..

[35] Corre J., Heébraud B., Bourin P. (2013). Concise Review: Growth Differentiation Factor 15 in Pathology: A Clinical Role?. Stem Cells Transl. Med..

[36] Mendez Fernandez A.B., FerreroGregori A., GarciaOsuna A., Mirabet-Perez S., Pirla-Buxo M.J., Cinca-Cuscullola J., Ordonez-Llanos J., Minguell E.R. (2020). Growth differentiation factor 15 as a mortality predictor in heart failure patients with nonreduced ejection fraction. ESC Heart Fail..

[37] Michowitz Y., Arbel Y., Wexler D., Sheps D., Rogowski O., Shapira I., Berliner S., Keren G., George J., Roth A. (2008). Predictive value of high sensitivity CRP in patients with diastolic heart failure. Int. J. Cardiol..

[38] Tromp J., Khan M.A.F., Klip I.T., Meyer S., de Boer R.A., Jaarsma T., Hillege H., van Veldhuisen D.J., van der Meer P., Voors A.A. (2017). Biomarker Profiles in Heart Failure Patients with Preserved and Reduced Ejection Fraction. J. Am. Heart Assoc..

[39] Arnar D.O., Mairesse G.H., Boriani G., Calkins H., Chin A., Coats A., Deharo J.C., Svendsen J.H., Heidbüchel H., Isa R. (2019). ESC Scientific Document Group; EHRA Scientific Documents Committee. Management of asymptomatic arrhythmias: A European Heart Rhythm Association (EHRA) consensus document, endorsed by the Heart Failure Association (HFA), Heart Rhythm Society (HRS), Asia Pacific Heart Rhythm Society (APHRS), Cardiac Arrhythmia Society of Southern Africa (CASSA), and Latin America Heart Rhythm Society (LAHRS). Europace.

[40] Adabag S., Patton K.K., Buxton A.E., Rector T.S., Ensrud K.E., Vakil K., Levy W.C., Poole J.E. (2017). Association of Implantable Cardioverter Defibrillators With Survival in Patients With and Without Improved Ejection Fraction: Secondary Analysis of the Sudden Cardiac Death in Heart Failure Trial. JAMA Cardiol..

[41] Lund L.H., Claggett B., Liu J., Lam C.S., Jhund P.S., Rosano G.M., Swedberg K., Yusuf S., Granger C.B., Pfeffer M.A. (2018). Heart failure with mid-range ejection fraction in CHARM: Characteristics, outcomes and effect of candesartan across the entire ejection fraction spectrum. Eur. J. Heart Fail..

[42] Solomon S.D., Claggett B., Lewis E.F., Desai A., Anand I., Sweitzer N.K., O’Meara E., Shah S.J., McKinlay S., Fleg J.L. (2016). TOPCAT Investigators. Influence of ejection fraction on outcomes and efficacy of spironolactone in patients with heart failure with preserved ejection fraction. Eur. Heart J..

[43] Solomon S.D., Vaduganathan M.L., Claggett B., Packer M., Zile M., Swedberg K., Rouleau J., Pfeffer M.A., Desai A., Lund L.H. (2020). Sacubitril/Valsartan Across the Spectrum of Ejection Fraction in Heart Failure. Circulation.

[44] Solomon S.D., McMurray J.J.V., Anand I.S., Ge J., Lam C.S., Maggioni A.P., Martinez F., Packer M., Pfeffer M.A., Pieske B. (2019). PARAGON-HF Investigators and Committees. Angiotensin-Neprilysin Inhibition in Heart Failure with Preserved Ejection Fraction. N. Engl. J. Med..

[45] Cleland J.G.F., Bunting K.V., Flather M.D., Altman D.G., Holmes J., Coats A.J., Manzano L., McMurray J.J., Ruschitzka F., van Veldhuisen D.J. (2018). Beta-blockers in Heart Failure Collaborative Group. Beta-blockers for heart failure with reduced, mid-range, and preserved ejection fraction: An individual patient-level analysis of double-blind randomized trials. Eur. Heart J..

[46] Abdul-Rahim A.H., Shen L., Rush C.J., Jhund P.S., Lees K.R., McMurray J.J. (2018). VICCTA-Heart Failure Collaborators. Effect of digoxin in patients with heart failure and mid-range (borderline) left ventricular ejection fraction. Eur. J. Heart Fail..

[47] Anker S.D., Butler J., Filippatos G., Ferreira J.P., Bocchi E., Böhm M., Brunner–La Rocca H.P., Choi D.J., Chopra V., Chuquiure-Valenzuela E. (2021). EMPEROR-Preserved Trial Investigators. Empagliflozin in Heart Failure with a Preserved Ejection Fraction. N. Engl. J. Med..

[48] Butler J., Filippatos G., Jamal Siddiqi T., Brueckmann M., Böhm M., Chopra V.K., Ferreira J.P., Januzzi J.L., Kaul S., Piña I.L. (2022). Empagliflozin, Health Status, and Quality of Life in Patients With Heart Failure and Preserved Ejection Fraction: The EMPEROR-Preserved Trial. Circulation.

[49] Packer M., Butler J., Zannad F., Filippatos G., Ferreira J.P., Pocock S.J., Carson P., Anand I., Doehner W., Haass M. (2021). Effect of Empagliflozin on Worsening Heart Failure Events in Patients With Heart Failure and Preserved Ejection Fraction: EMPEROR-Preserved Trial. Circulation.

[50] Mc Causland F.R., Claggett B.L., Vaduganathan M., Desai A., Jhund P., Vardeny O., Fang J.C., de Boer R.A., Docherty K.F., Hernandez A.F. (2024). Decline in Estimated Glomerular Filtration Rate After Dapagliflozin in Heart Failure With Mildly Reduced or Preserved Ejection Fraction: A Prespecified Secondary Analysis of the DELIVER Randomized Clinical Trial. JAMA Cardiol..

[51] Peikert A., Bart B.A., Vaduganathan M., Claggett B.L., Kulac I.J., Kosiborod M.N., Desai A.S., Jhund P.S., Lam C.S., Inzucchi S.E. (2024). Contemporary Use and Implications of Beta-Blockers in Patients With HfmrEF or HFpEF: The DELIVER Trial. JACC Heart Fail..

[52] Armstrong P.W., Pieske B., Anstrom K.J., Ezekowitz J., Hernandez A.F., Butler J., Lam C.S., Ponikowski P., Voors A.A., Jia G. (2020). VICTORIA Study Group. Vericiguat in Patients with Heart Failure and Reduced Ejection Fraction. N. Engl. J. Med..

[53] Chung M.K., Patton K.K., Lau C.P., Forno A.R.D., Al-Khatib S.M., Arora V., Birgersdotter-Green U.M., Cha Y.-M., Chung E.H., Cronin E.M. (2023). 2023 HRS/APHRS/LAHRS guideline on cardiac physiologic pacing for the avoidance and mitigation of heart failure. Heart Rhythm..

[54] Shroff J.P., Chandh Raja D., Tuan L.Q., Abhilash S.P., Mehta A., Abhayaratna W.P., Sanders P., Pathak R.K. (2024). Efficacy of left bundle branch area pacing versus biventricular pacing in patients treated with cardiac resynchronization therapy: Select site—Cohort study. Heart Rhythm..

[55] Abhilash S.P., Raja D.C., Stolcman S., Yi D.S., Rahman M., Tan R., Mahajan A., Lau D.H., Abhayaratna W.P., Sanders P. (2022). Computerized tomography image correlation of His bundle/deep septal pacing location and outcomes: An analysis from the Canberra HIs bundle/deep septal Pacing Study (CHIPS). J. Interv. Card. Electrophysiol..

[56] Raja D.C., Samarawickrema I., Menon S.K., Singh R., Mehta A., Tuan L.Q., Pandurangi U., Jain S., Callans D.J., Marchlinski F.E. (2024). Characteristics of the phenotype of mixed cardiomyopathy in patients with implantable cardioverter-defibrillators. J. Interv. Card. Electrophysiol..

[57] Shah S.J., Borlaug B.A., Chung E.S., Cutlip D., Debonnaire P., Fail P.S., Gao Q., Hasenfuß G., Kahwash R., Kaye D.M. (2022). REDUCE LAP-HF II investigators. Atrial shunt device for heart failure with preserved and mildly reduced ejection fraction (REDUCE LAP-HF II): A randomised, multicentre, blinded, sham-controlled trial. Lancet.

[58] Feldman T., Mauri L., Kahwash R., Litwin S., Ricciardi M.J., Van Der Harst P., Penicka M., Fail P.S., Kaye D.M., Petrie M.C. (2018). REDUCE LAP-HF I Investigators and Study Coordinators. Transcatheter Interatrial Shunt Device for the Treatment of Heart Failure With Preserved Ejection Fraction (REDUCE LAP-HF I [Reduce Elevated Left Atrial Pressure in Patients With Heart Failure]): A Phase 2, Randomized, Sham-Controlled Trial. Circulation.

[59] Shah S.J., Feldman T., Ricciardi M.J., Kahwash R., Lilly S., Litwin S., Nielsen C.D., Van Der Harst P., Hoendermis E., Penicka M. (2018). One-Year Safety and Clinical Outcomes of a Transcatheter Interatrial Shunt Device for the Treatment of Heart Failure With Preserved Ejection Fraction in the Reduce Elevated Left Atrial Pressure in Patients With Heart Failure (REDUCE LAP-HF I) Trial: A Randomized Clinical Trial. JAMA Cardiol..

[60] Packer D.L., Mark D.B., Robb R.A., Monahan K.H., Bahnson T.D., Poole J.E., Noseworthy P.A., Rosenberg Y.D., Jeffries N., Mitchell L.B. (2019). Effect of Catheter Ablation vs Antiarrhythmic Drug Therapy on Mortality, Stroke, Bleeding, and Cardiac Arrest Among Patients With Atrial Fibrillation: The CABANA Randomized Clinical Trial. JAMA.

[61] Lopes R.D., Alexander K.P., Stevens S.R., Reynolds H.R., Stone G.W., Piña I.L., Rockhold F.W., Elghamaz A., Lopez-Sendon J.L., Farsky P.S. (2020). Initial Invasive Versus Conservative Management of Stable Ischemic Heart Disease in Patients With a History of Heart Failure or Left Ventricular Dysfunction: Insights From the ISCHEMIA Trial. Circulation.

[62] Ponikowski P., van Veldhuisen D.J., Comin-Colet J., Ertl G., Komajda M., Mareev V., McDonagh T., Parkhomenko A., Tavazzi L., Levesque V. (2015). CONFIRM-HF Investigators. Beneficial effects of long-term intravenous iron therapy with ferric carboxymaltose in patients with symptomatic heart failure and iron deficiency. Eur. Heart J..

[63] Doukky R., Mangla A., Ibrahim Z., Poulin M.-F., Avery E., Collado F.M., Kaplan J., Richardson D., Powell L.H. (2016). Impact of Physical Inactivity on Mortality in Patients With Heart Failure. Am. J. Cardiol..

[64] Kawaji T., Shizuta S., Aizawa T., Yamagami S., Kato M., Yokomatsu T., Miki S., Ono K., Kimura T. (2021). Impact of catheter ablation for atrial fibrillation on cardiac disorders in patients with coexisting heart failure. ESC Heart Fail..

[65] Stevenson W. (2011). Ventricular Arrhythmias. Goldman’s Cecil Medicine.

[66] Raja D.C., Samarawickrema I., Das S., Mehta A., Tuan L., Jain S., Dixit S., Marchlinski F., Abhayaratna W.P., Sanders P. (2022). Long-term mortality in heart failure with mid-range ejection fraction: Systematic review and meta-analysis. ESC Heart Fail..

[67] Löfman I., Szummer K., Dahlström U., Jernberg T., Lund L.H. (2017). Associations with and prognostic impact of chronic kidney disease in heart failure with preserved, mid-range, and reduced ejection fraction. Eur. J. Heart Fail..

[68] Koh A.S., Tay W.T., Teng T.H.K., Vedin O., Benson L., Dahlstrom U., Savarese G., Lam C.S., Lund L.H. (2017). A comprehensive population-based characterization of heart failure with mid-range ejection fraction. Eur. J. Heart Fail..

